# Cemiplimab-Induced Hyperosmolar Hyperglycemic State With Concurrent Diabetic Ketoacidosis in a Patient Receiving Treatment for Cutaneous Squamous Cell Carcinoma

**DOI:** 10.7759/cureus.60565

**Published:** 2024-05-18

**Authors:** Alexander Pyronneau, Kelvin Noronha, Amanda Zucker, Rachel Kennett, Parth Desai

**Affiliations:** 1 Internal Medicine, HCA Healthcare/USF Morsani College of Medicine GME: HCA Florida Trinity Hospital, Trinity, USA; 2 Critical Care Medicine, HCA Healthcare/USF Morsani College of Medicine GME: HCA Florida Trinity Hospital, Trinity, USA

**Keywords:** new-onset diabetes mellitus, hyperglycemic hyperosmolar non-ketotic syndrome, dka, cutaneous squamous cell carcinoma (cscc), cemiplimab

## Abstract

The immune checkpoint inhibitor (ICI) cemiplimab is a human monoclonal antibody used in the treatment of locally advanced and metastatic cutaneous squamous cell carcinoma (CSCC) not amenable to surgery or radiation therapy. Although cemiplimab shows excellent efficacy with a good tolerability profile, it can cause side effects, including potentially life-threatening endocrinopathies. We discuss the case of a 77-year-old Caucasian female with CSCC treated with only three cycles of cemiplimab who presented with altered mental status and was found to have severe hyperglycemia, hyperosmolarity, ketonemia, glucosuria, and ketonuria concerning for hyperosmolar hyperglycemic syndrome (HHS) with concurrent diabetic ketoacidosis (DKA). The patient made a rapid recovery in the hospital while on standard therapies for HHS/DKA and cemiplimab was discontinued upon discharge. While there have been reports of cemiplimab-induced DKA, to our knowledge, this is the first reported case of cemiplimab-induced HHS-DKA. This report aims to shed light on cemiplimab-induced HHS-DKA and to underscore the need to elucidate the molecular mechanisms underlying ICI-induced diabetes mellitus (ICI-DM).

## Introduction

Cutaneous squamous cell carcinoma (CSCC) is the second most common nonmelanoma skin cancer, which accounts for 20% of all skin cancers [1]. While most CSCCs are treated with surgical intervention, approximately 5% display an aggressive phenotype that is not amenable to surgery or radiation therapy [2]. This paved the way for exploring immunotherapy options to treat advanced cases of CSCC. Thus, in 2018, cemiplimab, a human monoclonal antibody that targets the programmed cell death receptor-1 (PD-1), became the first immune checkpoint inhibitor (ICI) approved by the Food and Drug Administration (FDA) and the European Medicines Agency for the treatment of locally advanced CSCC (laCSCC) and metastatic CSCC (mCSCC) [3]. The use of cemiplimab in laCSCC and mCSCC has shown excellent objective response rates with good tolerability [4-6]. Although cemiplimab shows good efficacy in patients with CSCC, it has been associated with side effects such as fatigue, diarrhea, anemia, rash, pruritus, musculoskeletal pain, pneumonia, and pancreatitis [2]. On rare occasions, cemiplimab can give rise to potentially life-threatening endocrinopathies such as diabetic ketoacidosis (DKA) [2].

We present the case of a 77-year-old Caucasian female with CSCC who, after receiving only three cycles of cemiplimab, presented with altered mental status, manifesting severe hyperglycemia, hyperosmolarity, ketonemia, glucosuria, and ketonuria concerning for hyperglycemic hyperosmolar syndrome (HHS) with concurrent DKA. The patient responded rapidly to standard therapies for HHS/DKA in the acute care setting, and cemiplimab was discontinued upon discharge. To our knowledge, this is the first reported case of cemiplimab-induced HHS with concurrent DKA (HHS-DKA). Our finding that HHS-DKA developed shortly after the initiation of cemiplimab for CSCC highlights the possibility that ICIs may play a critical role in various mechanisms of pancreatic dysfunction.

## Case presentation

A 77-year-old Caucasian female presented to the emergency department with a three-day history of altered mental status in the setting of lethargy, decreased appetite, weight loss, nausea, nonbilious emesis, abdominal pain, and lightheadedness. She had a past medical history of refractory CSCC of the left foot and bilateral lower extremities, paroxysmal atrial fibrillation, hyperlipidemia, and hypothyroidism.

**Figure 1:**
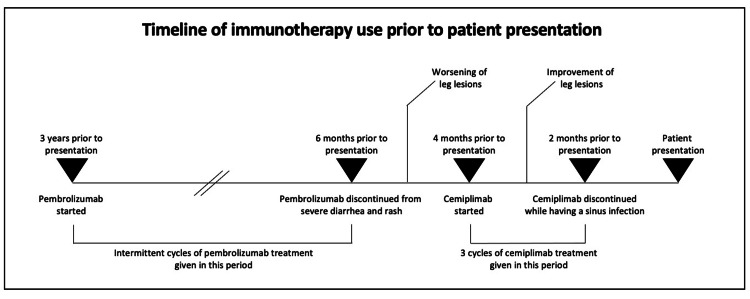
Timeline of immunotherapy use before the current presentation Three years before presentation, the patient had undergone intermittent cycles of pembrolizumab for two and a half years, which had ceased six months before admission due to severe diarrhea and rash. Subsequently, cemiplimab had been started four months before admission and administered for a total of three cycles, ending two months before the presentation

The patient had been initially diagnosed with CSCC of the left foot 16 years before this presentation and had undergone surgical resection. Fifteen years before this presentation, she had received radiation therapy due to lesion recurrence. A biopsy of a newly developing left heel ulcer three years before had revealed a recurrence of invasive CSCC for which surgery and radiation were not viable. The patient had been started on the ICI pembrolizumab three years before and completed several intermittent cycles of treatment over a two-and-a-half-year period; however, pembrolizumab had been ultimately discontinued six months before at the patient’s request due to severe diarrhea and rash that had limited the frequency of treatment (Figure 1). Due to worsening skin lesions, the ICI cemiplimab had been started four months before presentation and was dosed monthly due to persistent diarrhea. Since starting cemiplimab, the patient had seen an improvement in her leg lesions. Of note, her gastrointestinal symptoms had responded to prednisone and infliximab. The patient had ultimately received three cycles of cemiplimab treatment, which had continued until two months before this presentation when the patient elected to stop treatment due to a severe sinus infection (Figure 1).

**Figure 2:**
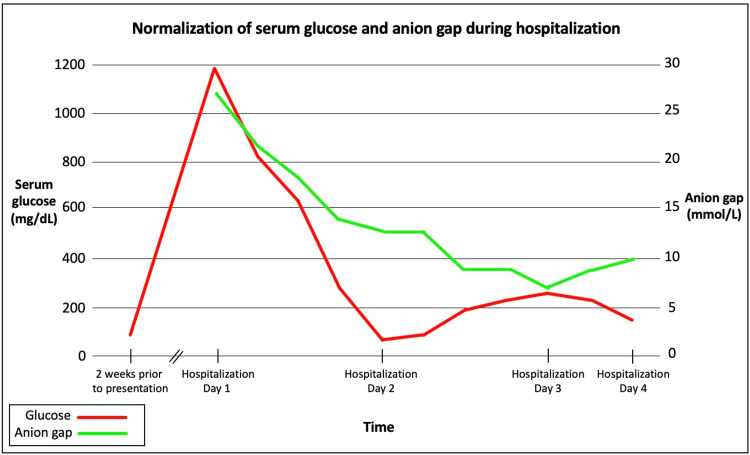
Rapid elevation and subsequent normalization of serum glucose and anion gap during hospitalization Graphical representation showing normal serum glucose two weeks before patient presentation, which rapidly increased to 1186 mg/dL on hospital day 1 and normalized on hospital day 2 (red line). Of note, the anion gap, which was elevated on patient presentation (27 mmol/L) normalized on hospital day 2 (green line)

On arrival, the patient was tachypneic and found to be in atrial fibrillation (Afib) with rapid ventricular response (RVR) with a heart rate of 160 beats per minute. She was immediately started on a diltiazem drip to manage her Afib-RVR in the emergency room. Physical examination showed cutaneous lesions on the bilateral lower extremities and left foot. Dry mucous membranes were suggestive of systemic dehydration. Laboratory studies revealed a glucose level of 1186 mg/dL (Figure 2; outpatient records showed that fasting blood glucose had been 84 mg/dL two weeks before presentation), a hemoglobin A1C (HbA1C) of 8.3%, an elevated serum beta-hydroxybutyrate of 71 mg/dL indicative of ketonemia and urinalysis significant for glucosuria and ketonuria. The basal metabolic panel showed a sodium of 128 mmol/L with corrected sodium of 145 mmol/L, potassium of 3.7 mmol/L, chloride of 86 mmol/L, bicarbonate of 15 mmol/L, and an elevated anion gap of 27 mmol/L consistent with DKA. The calculated serum osmolality on presentation was 338 mOsm/kg, which raised suspicion for concurrent HHS.

The patient was also found to have an acute kidney injury (BUN of 44 mg/dL and creatinine of 2.5 mg/dL with a baseline creatinine of 1.0 mg/dL) as well as hyperlactatemia (lactic acid: 5.4 mmol/L) likely secondary to severe dehydration. Of note, anti-glutamic acid decarboxylase (GAD) antibodies were negative. The patient was admitted directly to the ICU and underwent aggressive treatment for HHS-DKA in the setting of dehydration. Per standardized protocol, normal saline was started with an insulin drip and electrolytes were replenished as needed. Within one day, normalization of the anion gap, glucose, bicarbonate, and lactic acid were achieved, and the patient’s mental status returned to baseline (Figure 2, Table 1). The patient’s kidney function returned to baseline on hospital day 4. The insulin drip was discontinued, the patient was started on long-acting and short-acting subcutaneous insulin and was then downgraded to the medical floor from the ICU. The patient remained in the hospital for a few more days for adequate rate control of Afib and was eventually discharged in stable condition.

**Table 1:** Rapid elevation and subsequent normalization of serum glucose and anion gap during hospitalization Table 1, corresponding to Figure 2, highlights the trend of the patient’s serum glucose and anion gap during the first four days of hospitalization with normalization of serum glucose and anion gap on hospital day 2

Timeline	Serum glucose (mg/dL)	Anion gap (mmol/L)
Two weeks before the presentation	84	–
Hospitalization day 1	1186	27
	823	22
	639	18
	277	14
Hospitalization day 2	66	13
	83	13
	186	9
	231	9
Hospitalization day 3	259	7
	229	9
Hospitalization day 4	150	10
Reference range	74-106	7-16

## Discussion

The ICI cemiplimab is a human monoclonal antibody that targets PD-1 for the treatment of advanced CSCC. Since its FDA approval in 2018 for the treatment of CSCC, cemiplimab has been used in several cancers and has shown great promise with good tolerability [7]. Thus, as the use of cemiplimab has risen, the potential for side effects has also continued to rise. One of those rare side effects, which affects approximately 1% of patients [8], includes DKA. In this report, we presented a unique case of cemiplimab-induced HHS-DKA in a patient being treated for CSCC.

The patient had received only three cycles of cemiplimab, which had concluded two months before the current presentation. Two weeks before presentation, the patient’s fasting blood glucose had been 84 mg/dL, which elevated to 1186 mg/dL on presentation in the setting of hyperosmolarity, ketonemia, glucosuria, and ketonuria suggestive of HHS-DKA. Our findings align with those of other studies, as ICI-induced HHS-DKA have been shown to occur as early as a few weeks after the initiation of immunotherapy to up to one year after the discontinuation of treatment ranging from 2 to 21 cycles [9]. It is important to note that since the patient had taken the ICI pembrolizumab intermittently over a two-and-a-half-year period, we cannot completely rule out the possibility that pembrolizumab also contributed to the patient’s ICI-induced diabetes mellitus (ICI-DM).

Pembrolizumab-induced DM has been shown to occur up to one year after the initiation of treatment; however, most cases occur less than six months after the initiation of treatment [10-12]. Since our patient had ceased taking pembrolizumab six months before presentation, it is less likely that pembrolizumab was a contributing factor and that the patient’s ICI-DM was secondary only to cemiplimab use. To our knowledge, this is the first report of cemiplimab-induced HHS-DKA. Aggressive fluid hydration, IV insulin, and electrolyte repletion led to a rapid recovery of the patient’s clinical status and cemiplimab was discontinued after discharge. Our findings highlight an extremely rare yet underrecognized and underreported emergency in ICI-DM: HHS-DKA in type 1 DM (T1DM) induced by ICIs [9].

While the pathophysiology underlying DKA and HHS are distinct, growing evidence suggests an overlap of clinical findings in select cases. DKA typically occurs in T1DM whereas HHS typically occurs in T2DM [13]. In DKA, reduced insulin production due to various factors such as ICIs, infection, and autoimmunity leads to an increase in counter-regulatory hormones (growth hormone, catecholamines, glucagon, and cortisol), which triggers hepatic gluconeogenesis, glycogenolysis, and lipolysis [14]. Hepatic gluconeogenesis, glycogenolysis, and elevated counter-regulatory hormones cause hyperglycemia whereas lipolysis causes an increase in free fatty acids such that the hepatic metabolism of free fatty acids leads to the production of ketones and ketoacids [14]. Thus, DKA is characterized by hyperglycemia (blood glucose ≥250 mg/dL), ketonemia, and metabolic acidosis (pH ≤7.3 or bicarbonate ≤15 mEq/L) [15]. In HHS, insulin resistance due to various factors such as obesity, stress such as surgery, and an acute illness can also lead to an increase in counter-regulatory hormones, precipitating hyperglycemia. However, two major differences between HHS and DKA have been noted: insulin levels in HHS are high enough to inhibit lipolysis/ketogenesis, and dehydration caused by osmotic diuresis is more severe leading to serum hyperosmolality [13]. Thus, HHS is characterized by severe hyperglycemia (blood glucose ≥600 mg/dL), plasma osmolarity >320 mOsm/kg, and no appreciable ketonemia or metabolic acidosis [15].

Although DKA and HHS occur via two different mechanisms, it is estimated that nearly 30% of patients who present with severe hyperglycemic crises have a combination of HHS and DKA [16]. However, no accepted definition currently exists to identify patients presenting with HHS and DKA due to a lack of sufficient data regarding patients' clinical characteristics, frequency, and prognosis [13]. One recent study sought to define HHS-DKA as follows: plasma osmolarity ≥320 mOsm/kg, initial blood sugar ≥600 mg/dL, pH ≤7.3, and ketonuria and/or ketonemia [9]. Based on this current definition, our patient exhibited HHS-DKA induced by cemiplimab use. Although cemiplimab-induced DKA has been described before [17], our findings differ in that our patient displayed cemiplimab-induced HHS-DKA. This is significant because compared with ICI-DKA patients, ICI-induced HHS-DKA patients are prone to have higher hyperglycemia, significantly higher HbA1C levels, more likely to experience acute kidney injury, and more likely to have received chemotherapy before immunotherapy [9].

**Figure 3:**
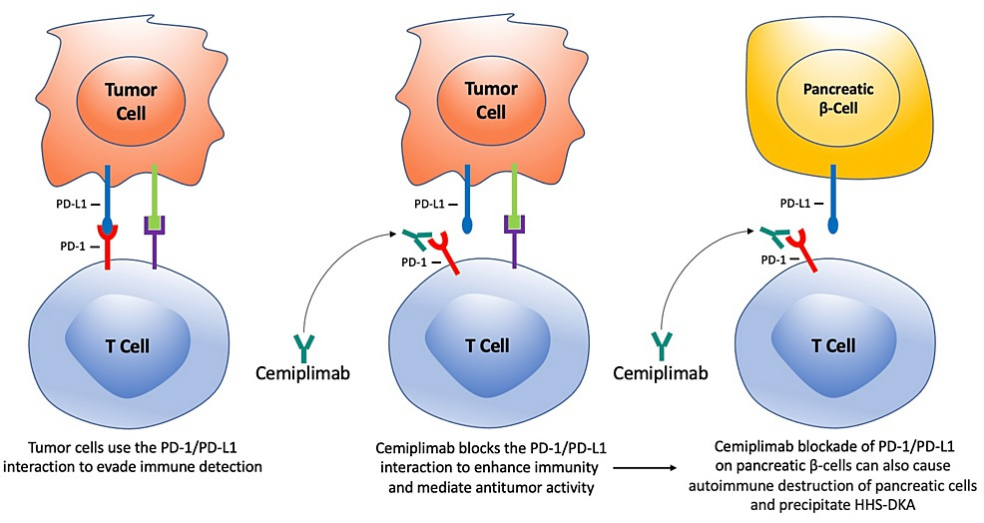
Proposed mechanism of cemiplimab-induced HHS-DKA T-cells express PD-1 and interact with cells expressing PD-L1 to prevent autoimmunity. Tumor cells can express PD-L1 and use the PD-1/PD-L1 interaction with T cells to evade immune system detection. Cemiplimab blocks the PD-1/PD-L1 interaction to enhance immune function and mediate antitumor activity. However, the PD-1/PD-L1 interaction is blocked between T cells and pancreatic β-cells potentially causing autoimmune destruction of pancreatic β-cells and thus precipitating HHS-DKA HHS-DKA: hyperglycemic hyperosmolar syndrome-diabetic ketoacidosis; PD-1: programmed cell death receptor-1; PD-L1: programmed death-ligand 1 The depicted figure was created by the authors

Although ICI-DM is well established, the pathophysiology underlying this phenomenon remains incompletely understood. PD-1 is expressed in T cells and interacts with programmed cell death-ligand 1 (PD-L1) and PD-L2 to inhibit T lymphocyte activation and prevent autoimmunity [18]. PD-L1 is upregulated in solid tumors and expressed on cells in the tumor microenvironment [18]. Tumor cells expressing PD-L1 can interact with T cells expressing PD-1 and the PD-1/PD-L1 interaction allows for tumor cells to evade detection by the immune system (Figure 3) [19]. ICIs, such as cemiplimab, block the PD-1/PD-L1 interaction to enhance immune function and mediate antitumor activity [20, 21]. Of note, pancreatic islet cells also express PD-L1 receptors to avoid immune-mediated destruction by T cells [12]. Moreover, inhibition of the PD-1/PD-L1 interaction accelerates diabetes in nonobese diabetic mice [22] and PD-L1 overexpression in beta cells prevents diabetes in these mice [23]. Importantly, patients with T1DM have reduced PD-1 expression in CD4+ T cells, indicating that CD4+ T cell expression may contribute to T1DM [24]. Therefore, a plausible scenario in our patient is that cemiplimab, while blocking the PD-1/PD-L1 interaction to enhance immune function and mediate antitumor activity as manifested by the patient’s improvement in her skin lesions, also caused autoimmune destruction of pancreatic islet cells, thereby triggering HHS-DKA (Figure 3).

It is important to note that other factors unique to ICI-DM may have contributed to the patient’s cemiplimab-induced HHS-DKA. In contrast to spontaneous T1DM, in ICI-DM, patients exhibit an almost complete absence of insulin-positive cells suggestive of more severe and rapid destruction of pancreatic beta cells [25]; 40-50% of individuals have detectable islet auto-antibodies at diagnosis (compared to 85-90% in spontaneous T1DM) [8,26], and patients are typically much older [10]. Genetic factors such as human leukocyte antigen (HLA)-DR4 have also been implicated in ICI-DM development [27]. Finally, ICI-DM may be associated with exocrine pancreatic inflammation as one-third of patients exhibit elevated amylase and lipase [8]. Collectively, some of these factors may have also contributed to our patient’s cemiplimab-induced HHS-DKA.

If a life-threatening immune-related adverse event (irAE) occurs, the ICI that initially caused it may need to be temporarily or permanently stopped. Some data suggest that the incidence of irAEs could either indicate a response to the cancer or that the ICI is not working and must be stopped [28]. Since our patient’s skin lesions improved on cemiplimab before the irAE, the irAE could have indicated a response to the cancer. However, since the patient permanently stopped cemiplimab, lesion recurrence of CSCC is highly likely given her clinical history. The current treatment strategies for irAEs include steroids to mitigate ongoing inflammation, immunosuppressants as second-line agents if patients do not respond to steroids, and supportive management (e.g., anti-inflammatories for ICI-arthritis, antidiarrheal agents for ICI-colitis, and antiarrhythmic therapy for ICI-myocarditis) [11].

Moreover, an important concern is an inability to de-escalate insulin therapy after ICI-DM occurs, as seen in our patient, which may indicate that immunotherapy-mediated beta cell destruction is irreversible [29]. Thus, an important future direction involves preventing or even reversing ICI-DM secondary to PD-1/PD-L1 disruption. Studies have sought to address this by utilizing human adipose-derived mesenchymal stem cells [30] and a JAK1/JAK2 inhibitor, with promising results [31,32]. For instance, DM was induced in nonobese diabetic mice using a PD-L1 inhibitor, and a JAK1/JAK2 inhibitor, which led to reduced insulitis secondary to PD-L1 treatment, reduced islet T-cell proliferation, and, significantly, prevented or reversed diabetes secondary to PD-L1 treatment [31]. In the same study, after immune checkpoint blockade, the JAK1/JAK2 inhibitor did not reverse or abrogate antitumor effects. Because JAK inhibitors, which are FDA-approved for rheumatoid arthritis and myelofibrosis, are generally well tolerated [33, 34], future research should test whether agents such as JAK inhibitors could prevent or even reverse ICI-DM in patients.

An important clinical practice moving forward should focus on educating patients and healthcare providers about hyperglycemic symptoms, HHS, DKA, and HHS-DKA at the time of cemiplimab initiation [35]. Fasting or random glucose and HbA1C should also be monitored at each cycle of cemiplimab therapy and when patients become hyperglycemic or develop symptoms [12]. If HHS, DKA, or HHS-DKA is confirmed, it should be managed according to institutional guidelines [35]. The severity of the ICI-DM will dictate whether the patient needs to stop treatment temporarily or permanently. Ultimately, with the growing use of cemiplimab and other ICIs in treating malignancies, more research is necessary regarding the pathophysiology, diagnostic criteria, treatment strategy, and prognosis of irAEs.

## Conclusions

Cemiplimab is an ICI used in the treatment of advanced CSCC. A rare yet potentially life-threatening side effect of cemiplimab use is ICI-induced DM (ICI-DM), which can manifest as HHS combined with DKA. We aimed to shed more light on various aspects of cemiplimab-induced HHS-DKA and to underscore the necessity to elucidate the molecular mechanisms underlying ICI-DM, which will in turn guide future treatment paradigms to help prevent or even reverse ICI-DM.
